# Supplementary material for: Antipsychotic drugs selectively decorrelate long-range interactions in deep cortical layers
Source: eLife. 2024 Apr 5;12:RP86805. doi: 10.7554/eLife.86805 (PMC10997332; doi:10.7554/eLife.86805)
Supplement: Supplementary file 1. — Note, for Figure 1L and Figure 1—figure supplement 4J, the significance thresholds were corrected for multiple comparisons (see Methods). [file elife-86805-supp1.docx]

Supplementary File 1

| Figure reference | Description  (N_1_ vs N_2_) | Test | N_1_ (mice) | N_2_ (mice) | Unit | P-value |
| --- | --- | --- | --- | --- | --- | --- |
| Figure 1F | Number of closed loop locomotion onsets in C57BL/6 mice | - | 907 (6) | - | Onsets |  |
| Figure 1G | Number of open loop locomotion onsets in C57BL/6 mice | - | 598 (6) | - | Onsets |  |
| Figure 1H | Number of open loop visual flow onsets in C57BL/6 mice | - | 416 (6) | - | Onsets |  |
| Figure 1I | Number of closed loop locomotion onsets in *Tlx3*-Cre x Ai148 mice | - | 1919 (15) | - | Onsets |  |
| Figure 1J | Number of open loop locomotion onsets in *Tlx3*-Cre x Ai148 mice | - | 1125 (15) | - | Onsets |  |
| Figure 1K | Number of open loop visual flow onsets in *Tlx3*-Cre x Ai148 mice | - | 1189 (15) | - | Onsets |  |
| Figure 1L | *Tlx3* vs C57BL/6 | Bootstrap | 12 (15) | 12 (6) | Regions | <10^-4^ |
|  | *Tlx3* vs *Emx1* | Bootstrap | 12 (15) | 12 (4) | Regions | 0.00135 |
|  | *Tlx3* vs *Cux2* | Bootstrap | 12 (15) | 12 (4) | Regions | <10^-4^ |
|  | *Tlx3* vs *Scnn1a* | Bootstrap | 12 (15) | 12 (7) | Regions | <10^-4^ |
|  | *Tlx3* vs *Ntsr1* | Bootstrap | 12 (15) | 12 (3) | Regions | 0.4541 |
|  | *Tlx3* vs *PV* | Bootstrap | 12 (15) | 12 (2) | Regions | <10^-4^ |
|  | *Tlx3* vs *VIP* | Bootstrap | 12 (15) | 12 (6) | Regions | 0.0001 |
|  | *Tlx3* vs *SST* | Bootstrap | 12 (15) | 12 (5) | Regions | 0.0131 |
|  | ANOVA | ANOVA | 108 (54) | N/A | Regions | < 10^-5^ |
| Figure 2B | V1 |  |  |  |  |  |
|  | Closed loop vs open loop | Hierachical bootsstrap | 308 (6) | 316 (6) | Recording session | 0.9856 |
|  | Closed loop vs dark | Hierachical bootsstrap | 308 (6) | 68 (6) | Recording session | 0.9631 |
|  | Open loop vs dark | Hierachical bootsstrap | 316 (6) | 68 (6) | Recording session | 0.8968 |
|  | RSC |  |  |  |  |  |
|  | Closed loop vs open loop | Hierachical bootsstrap | 308 (6) | 316 (6) | Recording session | 0.3423 |
|  | Closed loop vs dark | Hierachical bootsstrap | 308 (6) | 68 (6) | Recording session | 0.1492 |
|  | Open loop vs dark | Hierachical bootsstrap | 316 (6) | 68 (6) | Recording session | 0.2362 |
|  | V2am |  |  |  |  |  |
|  | Closed loop vs open loop | Hierachical bootsstrap | 308 (6) | 316 (6) | Recording session | 0.4576 |
|  | Closed loop vs dark | Hierachical bootsstrap | 308 (6) | 68 (6) | Recording session | 0.4694 |
|  | Open loop vs dark | Hierachical bootsstrap | 316 (6) | 68 (6) | Recording session | 0.4961 |
|  | M1 |  |  |  |  |  |
|  | Closed loop vs open loop | Hierachical bootsstrap | 308 (6) | 316 (6) | Recording session | 0.4417 |
|  | Closed loop vs dark | Hierachical bootsstrap | 308 (6) | 68 (6) | Recording session | 0.06 |
|  | Open loop vs dark | Hierachical bootsstrap | 316 (6) | 68 (6) | Recording session | 0.0788 |
|  | A24b |  |  |  |  |  |
|  | Closed loop vs open loop | Hierachical bootsstrap | 308 (6) | 316 (6) | Recording session | 0.8310 |
|  | Closed loop vs dark | Hierachical bootsstrap | 308 (6) | 68 (6) | Recording session | 0.2194 |
|  | Open loop vs dark | Hierachical bootsstrap | 316 (6) | 68 (6) | Recording session | 0.0846 |
|  | M2 |  |  |  |  |  |
|  | Closed loop vs open loop | Hierachical bootsstrap | 308 (6) | 316 (6) | Recording session | 0.4593 |
|  | Closed loop vs dark | Hierachical bootsstrap | 308 (6) | 68 (6) | Recording session | 0.2373 |
|  | Open loop vs dark | Hierachical bootsstrap | 316 (6) | 68 (6) | Recording session | 0.2674 |
| Figure 2D | V1 |  |  |  |  |  |
|  | Closed loop vs open loop | Hierachical bootsstrap | 420 (15) | 394 (15) | Recording session | <10^-4^ |
|  | Closed loop vs dark | Hierachical bootsstrap | 420 (15) | 194 (15) | Recording session | <10^-4^ |
|  | Open loop vs dark | Hierachical bootsstrap | 394 (15) | 194 (15) | Recording session | 0.2103 |
|  | RSC |  |  |  |  |  |
|  | Closed loop vs open loop | Hierachical bootsstrap | 420 (15) | 394 (15) | Recording session | <10^-4^ |
|  | Closed loop vs dark | Hierachical bootsstrap | 420 (15) | 194 (15) | Recording session | 0.0001 |
|  | Open loop vs dark | Hierachical bootsstrap | 394 (15) | 194 (15) | Recording session | 0.0021 |
|  | V2am |  |  |  |  |  |
|  | Closed loop vs open loop | Hierachical bootsstrap | 420 (15) | 394 (15) | Recording session | <10^-4^ |
|  | Closed loop vs dark | Hierachical bootsstrap | 420 (15) | 194 (15) | Recording session | <10^-4^ |
|  | Open loop vs dark | Hierachical bootsstrap | 394 (15) | 194 (15) | Recording session | 0.1337 |
|  | M1 |  |  |  |  |  |
|  | Closed loop vs open loop | Hierachical bootsstrap | 420 (15) | 394 (15) | Recording session | <10^-4^ |
|  | Closed loop vs dark | Hierachical bootsstrap | 420 (15) | 194 (15) | Recording session | 0.3115 |
|  | Open loop vs dark | Hierachical bootsstrap | 394 (15) | 194 (15) | Recording session | 0.0002 |
|  | A24b |  |  |  |  |  |
|  | Closed loop vs open loop | Hierachical bootsstrap | 420 (15) | 394 (15) | Recording session | <10^-4^ |
|  | Closed loop vs dark | Hierachical bootsstrap | 420 (15) | 194 (15) | Recording session | 0.3511 |
|  | Open loop vs dark | Hierachical bootsstrap | 394 (15) | 194 (15) | Recording session | 0.0001 |
|  | M2 |  |  |  |  |  |
|  | Closed loop vs open loop | Hierachical bootsstrap | 420 (15) | 394 (15) | Recording session | 0.0005 |
|  | Closed loop vs dark | Hierachical bootsstrap | 420 (15) | 194 (15) | Recording session | 0.2686 |
|  | Open loop vs dark | Hierachical bootsstrap | 394 (15) | 194 (15) | Recording session | 0.0156 |
| Figure 3B | Number of mismatch onsets in C57BL/6 mice | - | 2512 (6) | - | Onsets | - |
| Figure 3C | Number of grating onsets in C57BL/6 mice | - | 1858 (6) | - | Onsets | - |
| Figure 3E | Number of mismatch onsets in *Tlx3*-Cre x Ai148 mice | - | 3297 (15) | - | Onsets | - |
| Figure 3F | Number of mismatch onsets in *Tlx3*-Cre x Ai148 mice | - | 5318 (15) | - | Onsets | - |
| Figure 4A | Number of closed loop locomotion onset in clozapine treated C57BL/6 mice | - | 273 (4) | - | Onsets |  |
| Figure 4A, inset | Number of closed loop locomotion onset in naïve C57BL/6 mice | - | 720 (4) | - | Onsets | - |
| Figure 4B | Number of open loop locomotion onset in clozapine treated C57BL/6 mice | - | 145 (4) | - | Onsets | - |
| Figure 4B, inset | Number of open loop locomotion onset in naive C57BL/6 mice | - | 570 (4) | - | Onsets | - |
| Figure 4C | Number of open loop visual flow onsets in clozapine treated C57BL/6 mice that were not locomoting | - | 99 (4) | - | Onsets | - |
| Figure 4C, inset | Number of open loop visual flow onsets in naïve C57BL/6 mice that were not locomoting | - | 404 (4) | - | Onsets | - |
| Figure 4D | Number of closed loop locomotion onset in clozapine treated *Tlx3*-Cre x Ai148 mice | - | 707 (5) | - | Onsets | - |
| Figure 4D, inset | Number of closed loop locomotion onset in naive *Tlx3*-Cre x Ai148 mice | - | 1101 (5) | - | Onsets | - |
| Figure 4E | Number of open loop locomotion onset in clozapine treated *Tlx3*-Cre x Ai148 mice | - | 350 (5) | - | Onsets | - |
| Figure 4E, inset | Number of open loop locomotion onset in naive *Tlx3*-Cre x Ai148 mice | - | 348 (5) | - | Onsets | - |
| Figure 4F | Number of open loop visual flow onsets in clozapine treated *Tlx3*-Cre x Ai148 mice that were not locomoting | - | 514 (5) | - | Onsets | - |
| Figure 4F, inset | Number of open loop visual flow onsets in naive *Tlx3*-Cre x Ai148 mice that were not locomoting | - | 568 (5) | - | Onsets | - |
| Figure 6C | Short-range vs no change | T-test | 122 (4) | - | Pairs of regions | 0.3791 |
|  | Long-range vs no change | T-test | 142 (4) | - | Pairs of regions | 0.9035 |
|  | Short-range vs long-range | Ranksum | 122 (4) | 142 (4) | Pairs of regions | 0.0511 |
| Figure 6F | Short-range vs no change | T-test | 182 (5) | - | Pairs of regions | <10^-5^ |
|  | Long-range vs no change | T-test | 148 (5) | - | Pairs of regions | <10^-10^ |
|  | Short-range vs long-range | Ranksum | 182 (5) | 148 (5) | Pairs of regions | <10^-4^ |
| Figure 7C | Short-range vs no change | T-test | 114 (3) | - | Pairs of regions | <10^-5^ |
|  | Long-range vs no change | T-test | 84 (3) | - | Pairs of regions | <10^-7^ |
|  | Short-range vs long-range | Ranksum | 114 (3) | 84 (3) | Pairs of regions | 0.0347 |
| Figure 7F | Short-range vs no change | T-test | 114 (3) | - | Pairs of regions | 0.7058 |
|  | Long-range vs no change | T-test | 84 (3) | - | Pairs of regions | 0.0435 |
|  | Short-range vs long-range | Ranksum | 114 (3) | 84 (3) | Pairs of regions | 0.0199 |
| Figure 7I | Short-range vs no change | T-test | 114 (3) | - | Pairs of regions | 0.4865 |
|  | Long-range vs no change | T-test | 84 (3) | - | Pairs of regions | 0.2184 |
|  | Short-range vs long-range | Ranksum | 114 (3) | 84 (3) | Pairs of regions | 0.1633 |
| Figure 8A | Number of mismatch onsets in naïve *Tlx3*-Cre x Ai148 mice | - | 2464 (11) | - | Onsets | - |
|  | Number of mismatch onsets in *Tlx3*-Cre x Ai148 mice treated with antipsychotic drugs | - | 2017 (11) | - | Onsets | - |
| Figure 8B | Number of grating onsets in naïve *Tlx3*-Cre x Ai148 mice | - | 3942 (11) | - | Onsets | - |
|  | Number of grating onsets in *Tlx3*-Cre x Ai148 mice treated with antipsychotic drugs | - | 1645 (11) | - | Onsets | - |
| Figure 1 – figure supplement 1A, left | Number of locomotion onsets in C57BL/6 mice that expressed GFP brain wide and were implanted with crystal skulls | - | 96 (3) | - | Onsets | - |
| Figure 1 – figure supplement 1A, right | Number of locomotion onsets in C57BL/6 mice that expressed GFP brain wide with clear skull cranial windows | - | 615 (5) | - | Onsets | - |
| Figure 1 – figure supplement 1B, left | Number of mismatch onsets in C57BL/6 mice that expressed GFP brain wide and were implanted with crystal skulls | - | 229 (3) | - | Onsets | - |
| Figure 1 – figure supplement 1B, right | Number of mismatch onsets in C57BL/6 mice that expressed GFP brain wide with clear skull cranial windows | - | 228 (5) | - | Onsets | - |
| Figure 1 – figure supplement 1C | Number of locomotion onsets in *Tlx3*-Cre mice that expressed GFP in L5 IT neurons | - | 1880 (7) | - | Onsets | - |
| Figure 1 – figure supplement 1D | Number of mismatch onsets in *Tlx3*-Cre mice that expressed GFP in L5 IT neurons | - | 451 (7) | - | Onsets | - |
| Figure 1 – figure supplement 3A | Number of closed loop locomotion onsets in *Tlx3*-Cre x Ai148 mice recorded with the widefield microscope | - | 1012 (7) | - | Onsets | - |
|  | Number of open loop locomotion onsets in *Tlx3*-Cre x Ai148 mice recorded with the widefield microscope | - | 678 (7) | - | Onsets | - |
| Figure 1 – figure supplement 3C | Number of mismatch onsets in *Tlx3*-Cre x Ai148 mice recorded with the widefield microscope | - | 1038 (7) | - | Onsets | - |
|  | Number of open loop halts in *Tlx3*-Cre x Ai148 mice recorded with the widefield microscope | - | 410 (7) | - | Onsets | - |
|  | Number of grating onsets in *Tlx3*-Cre x Ai148 mice recorded with the widefield microscope | - | 2014 (7) | - | Onsets | - |
| Figure 1 – figure supplement 4A | Number of closed loop locomotion onsets in *Emx1*-Cre mice | - | 438 (4) | - | Onsets | - |
|  | Number of open loop locomotion onsets in *Emx1*-Cre mice | - | 286 (4) | - | Onsets | - |
| Figure 1 – figure supplement 4B | Number of closed loop locomotion onsets in *Cux2*-CreERT2 x Ai148 mice | - | 415 (4) | - | Onsets | - |
|  | Number of open loop locomotion onsets in *Cux2*-CreERT2 x Ai148 mice | - | 433 (4) | - | Onsets | - |
| Figure 1 – figure supplement 4C | Number of closed loop locomotion onsets in *Scnn1a*-Cre x Ai148 mice | - | 839 (7) | - | Onsets | - |
|  | Number of open loop locomotion onsets in *Scnn1a*-Cre x Ai148 mice | - | 313 (7) | - | Onsets | - |
| Figure 1 – figure supplement 4D | Number of closed loop locomotion onsets in *Tlx3*-Cre x Ai148 mice | - | 1919 (15) | - | Onsets | - |
|  | Number of open loop locomotion onsets in *Tlx3*-Cre x Ai148 mice | - | 1125 (15) | - | Onsets | - |
| Figure 1 – figure supplement 4E | Number of closed loop locomotion onsets in *Ntsr1*-Cre x Ai148 mice | - | 368 (3) | - | Onsets | - |
|  | Number of open loop locomotion onsets in *Ntsr1*-Cre x Ai148 mice | - | 112 (3) | - | Onsets | - |
| Figure 1 – figure supplement 4F | Number of closed loop locomotion onsets in C57BL/6 mice | - | 907 (6) | - | Onsets | - |
|  | Number of open loop locomotion onsets in C57BL/6 mice | - | 598 (6) | - | Onsets | - |
| Figure 1 – figure supplement 4G | Number of closed loop locomotion onsets in *PV*-Cre x Ai148 mice | - | 236 (2) | - | Onsets | - |
|  | Number of open loop locomotion onsets in *PV*-Cre x Ai148 mice | - | 110 (2) | - | Onsets | - |
| Figure 1 – figure supplement 4H | Number of closed loop locomotion onsets in *VIP*-Cre x Ai148 mice | - | 618 (6) | - | Onsets | - |
|  | Number of open loop locomotion onsets in *VIP*-Cre x Ai148 mice | - | 376 (6) | - | Onsets | - |
| Figure 1 – figure supplement 4I | Number of closed loop locomotion onsets in *SST*-Cre x Ai148 mice | - | 747 (5) | - | Onsets | - |
|  | Number of open loop locomotion onsets in *SST*-Cre x Ai148 mice | - | 558 (5) | - | Onsets | - |
| Figure 1 – figure supplement 4J | *Tlx3* vs C57BL/6 | Bootstrap | 14 | 6 | V1 / Mice | <10^-4^ |
|  | *Tlx3* vs *Emx1* | Bootstrap | 14 | 4 | V1 / Mice | 0.0042 |
|  | *Tlx3* vs *Cux2* | Bootstrap | 14 | 3 | V1 / Mice | <10^-4^ |
|  | *Tlx3* vs *Scnn1a* | Bootstrap | 14 | 7 | V1 / Mice | 0.0042 |
|  | *Tlx3* vs *Ntsr1* | Bootstrap | 14 | 3 | V1 / Mice | 0.4087 |
|  | *Tlx3* vs *PV* | Bootstrap | 14 | 2 | V1 / Mice | <10^-4^ |
|  | *Tlx3* vs *VIP* | Bootstrap | 14 | 6 | V1 / Mice | 0.0021 |
|  | *Tlx3* vs *SST* | Bootstrap | 14 | 6 | V1 / Mice | 0.0032 |
|  | ANOVA | ANOVA | 54 | N/A | V1 / Mice | 0.0004 |
| Figure 4 – figure supplement 1A | Number of locomotion onsets in *Tlx3*-Cre mice | - | 2331 (7) | - | Onsets | - |
| Figure 4 – figure supplement 1C | Naive vs +1 h antipsy. | Ranksum | 21 | 22 | Mice | 0.4735 |
| Figure 4 – figure supplement 1D | C57BL/6 |  |  |  |  |  |
|  | Closed loop vs no change | T-test | 24 (4) | - | Regions | 0.0277 |
|  | Open loop vs no change | T-test | 24 (4) | - | Regions | 0.6384 |
|  | Dark vs no change | T-test | 24 (4) | - | Regions | <10^-4^ |
|  | *Tlx3* |  |  |  |  |  |
|  | Closed loop vs no change | T-test | 30 (5) | - | Regions | 0.0365 |
|  | Open loop vs no change | T-test | 30 (5) | - | Regions | 0.0004 |
|  | Dark vs no change | T-test | 30 (5) | - | Regions | 0.0104 |
| Figure 4 – figure supplement 1E | Number of closed loop locomotion onsets in *Tlx3*-Cre x Ai148 mice | - | 376 (3) | - | Onsets | - |
|  | Number of open loop locomotion onsets in *Tlx3*-Cre x Ai148 mice | - | 245 (3) | - | Onsets | - |
| Figure 5 – figure supplement 1E | Short-range vs no change | T-test | 204 (7) | - | Pairs of regions | 0.0006 |
|  | Long-range vs no change | T-test | 192 (7) | - | Pairs of regions | <10^-4^ |
|  | Short-range vs long-range | Ranksum | 204 (7) | 192 (7) | Pairs of regions | 0.2540 |
| Figure 5 – figure supplement 1H | Short-range vs no change | T-test | 93 (3) | - | Pairs of regions | 0.7884 |
|  | Long-range vs no change | T-test | 105 (3) | - | Pairs of regions | 0.1176 |
|  | Short-range vs long-range | Rank-sum | 93 (3) | 105 (3) | Pairs of regions | 0.2319 |
| Figure 6 – figure supplement 1C | Short-range vs no change | T-test | 114 (3) | - | Pairs of regions | <10^-4^ |
|  | Long-range vs no change | T-test | 84 (3) | - | Pairs of regions | <10^-4^ |
|  | Short-range vs long-range | Ranksum | 114 (3) | 84 (3) | Pairs of regions | <10^-4^ |
| Figure 6 – figure supplement 1D | Naive vs clozapine | Ranksum | 384108 (7) | 885596 | Pairs of neurons | <10^-4^ |
|  | Naive vs clozapine 1 site excl. | Ranksum | 384108 (7) | 831640 | Pairs of neurons | <10^-4^ |
| Figure 6 – figure supplement 2E | Short-range vs no change | T-test | 137 (4) | - | Pairs of regions | 0.0262 |
|  | Long-range vs no change | T-test | 127 (4) | - | Pairs of regions | 0.0019 |
|  | Short-range vs long-range | Ranksum | 137 (4) | 127 (4) | Pairs of regions | 0.9537 |
