# Supplementary material for: Antipsychotic drugs selectively decorrelate long-range interactions in deep cortical layers
Source: eLife. 2024 Apr 5;12:RP86805. doi: 10.7554/eLife.86805 (PMC10997332; doi:10.7554/eLife.86805)
Supplement: Supplementary file 2. [file elife-86805-supp2.docx]

Supplementary File 2

| **Nbr. mice** | **Genotype** | **Virus** | **Figures** |
| --- | --- | --- | --- |
| 3 | C57BL/6 | AAV-PHP.eB-hSyn1-jGCaMP7f | **1C, 1F-1H, 1L, 2A and 2B, 3A-3C, 4A-4C, 5A and 5B, 6A-6C, Figure 1 – figure supplement 2, Figure 1 – figure supplement 4F, Figure 1 – figure supplement 4J, Figure 4 – figure supplements 1B-1D, Figure 5 – figure supplements 1A and 1B** |
| 3 | C57BL/6 | AAV-PHP.eB-EF1α-GCaMP6s | **1F-1H, 1L, 2B, 3B and 3C, 4A-4C, 5A and 5B, 6A-6C, Figure 1 – figure supplement 2, Figure 1 – figure supplement 4F, Figure 1 – figure supplement 4J, Figure 4 – figure supplements 1B-1D** |
| 8 | C57BL/6 | AAV-PHP.eB-EF1α-eGFP | **Figure 1 – figure supplement 1** |
| 4 | *Emx1*-Cre | AAV-PHP.eB-DIO-EF1α-GCaMP6s | **1L, Figure 1 – figure supplements 4A and 4J** |
| 4 | *Cux2*-CreERT2 x Ai148 | - | **1L, Figure 1 – figure supplements 4B and 4J, Figure 6 – figure supplement 2** |
| 7 | *Scnn1a*-Cre x Ai148 | - | **1L, Figure 1 – figure supplements 4C and 4J** |
| 25 | *Tlx3*-Cre x Ai148 | - | **1D, 1I-1L, 2C and 2D, 3D-3F, 4D-4F, 5C and 5D, 6D-6F, 7, 8, Figure 1 – figure supplement 2, Figure 1 – figure supplement 3D, Figure 1 – figure supplements 4D and 4J, Figure 4 – figure supplements 1B-1F, Figure 5 – figure supplements 1F-1H, Figure 6 – figure supplement 1** |
| 7 | *Tlx3*-Cre | AAV-PHP.eB-EF1α-DIO-eGFP | **Figure 1 – figure supplements 1C and 1D, Figure 4 – figure supplement 1A, Figure 5 – figure supplements 1C-1E** |
| 3 | *Ntsr1*-Cre x Ai148 | - | **1L, Figure 1 – figure supplements 4E and 4J** |
| 2 | *PV*-Cre x Ai148 | - | **1L, Figure 1 – figure supplements 4G and 4J** |
| 6 | *VIP*-Cre x Ai148 | - | **1L, Figure 1 – figure supplements 4H and 4J** |
| 4 | *SST*-Cre | AAV-PHP.eB-DIO-EF1α-jGCaMP7f | **1L, Figure 1 – figure supplements 4I and 4J** |
| 1 | *SST*-Cre x Ai148 | - | **1L, Figure 1 – figure supplements 4I and 4J** |
